# Bile duct adenocarcinoma with minor micropapillary component: a case report

**DOI:** 10.1186/1757-1626-2-51

**Published:** 2009-01-14

**Authors:** Takeshi Kondo

**Affiliations:** 1 Division of Pathology (Molecular Pathology), Kobe University Graduate School of Medicine, Kobe, Japan

## Abstract

Invasive micropapillary carcinoma (IMPC) is defined as a carcinoma composed of small clusters of tumor cells lying within clear spaces which simulate lymphovascular channels. This histologic pattern has been described in various organs, including the breast, lung, urinary bladder, ovary, stomach, pancreas, and major salivary glands. Although rarely observed as a pure histologic component, IMPC is usually mixed with conventional carcinoma, and is therefore often referred to as carcinoma with a micropapillary component. IMPCs are invariably associated with a high degree of aggressiveness, extensive lymphovascular invasion, extensive lymph node metastases, and poor prognosis. Here a case of bile duct adenocarcinoma with minor micropapillary component is described.

## Introduction

Invasive micropapillary carcinoma (IMPC) is defined as a carcinoma composed of small clusters of tumor cells lying within clear spaces, and was proposed as a histological variant of invasive breast carcinoma with poor clinical prognosis [1]. This distinct histologic pattern has been described in various organs, including the urinary bladder, lung, ovary, colon, stomach, pancreas, and major salivary glands [2-9]. IMPC is usually mixed with otherwise conventional carcinoma [1], and is therefore often referred to as carcinoma with a micropapillary component. IMPCs are all invariably associated with a high degree of aggressiveness, extensive lymphovascular invasion, extensive lymph node metastases, and poor prognosis [1].

## Case presentation

**Figure 1:**
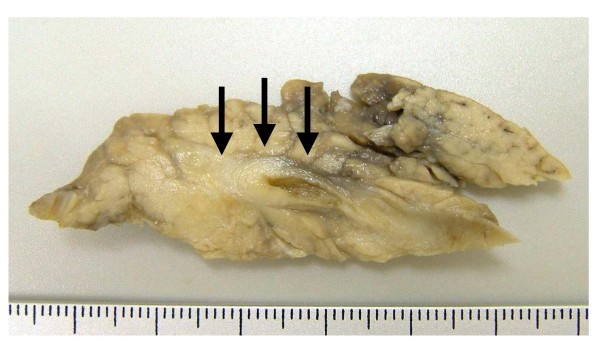
**Gross finding of the tumor**. The cancer was found (arrows) surrounding the inferior common bile duct.

**Figure 2:**
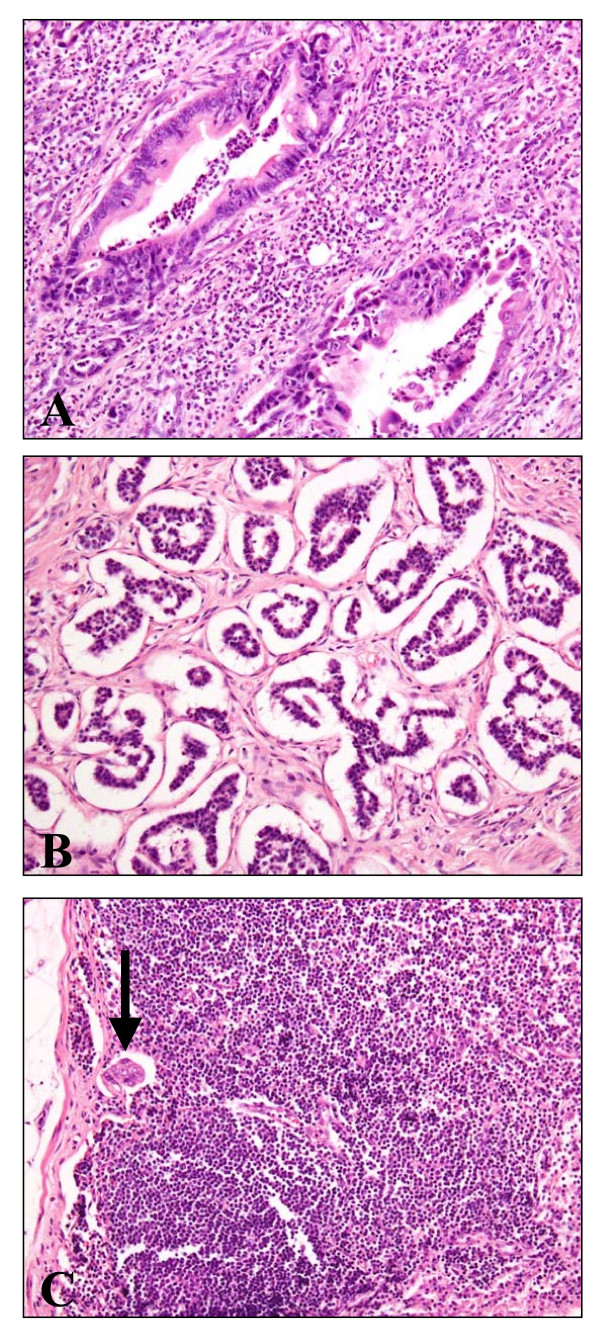
**Histology of the tumor (HE staining, ×200)**. **A**: conventional adenocarcinoma with intraepithelial, intraluminal, and stromal neutrophils. **B**: micropapillary component invading the Oddi's sphincter. **C**: micrometastasis in the regional lymph node (arrow).

A 75-year-old Japanese woman with inferior bile duct cancer presented. The patient had undergone pancreaticoduodenectomy for her tumor surrounding the inferior common bile duct, measuring 11 mm on cut surface (Fig. 1). Microscopically, it was composed of conventional adenocarcinoma with severe neutrophilic infiltration (Fig. 2A), and a small focus (2 mm in size) of micropapillary carcinoma was found in Oddi's sphincter (Fig. 2B). Carcinoma cells invaded the smooth muscle layer with foci of marked lymphatic invasion, although this lesion was small. The tumor was diagnosed as bile duct adenocarcinoma with a small amount of micropapillary carcinoma. Resected regional lymph nodes revealed micrometastasis of a few cancer cells in a micropapillary pattern only in one lymph node (Fig. 2C). The patient is alive and free of the recurrent disease at the latest followup (after 1 year since the operation).

## Discussion

IMPC has distinctive histologic features characterized by tufts of tumor cells arranged in pseudopapillary patterns without fibrovascular cores and surrounded by empty and clear spaces. This "inside-out growth" structure has been attributed to the rotation of cell polarization, whereby the stroma-facing (basal) surface of the cells acquires "apical" properties [1]. This inversion of cell polarity has been disclosed immunohistochemically by the outer membranous staining pattern of EMA only toward the stroma [1]. Compared with conventional carcinomas of similar size, hypothetically this reverse polarization in IMPC facilitates the secretion by tumor cells of molecules, such as metalloproteinases, known to be responsible for stromal and vascular invasion, permitting easier dissemination and a higher tendency for lymph node metastases [1].

All pancreatic micropapillary carcinoma reported arose from the ampullo-pancreatobiliary region (pancreatic head). Occurrence of the micropapillary component in the adenocarcinoma of gastrointestinal tract may be attributable to the continuous exposure of the mucosa to bile acids [6]. Taken together, it is suggested that the effect of bile juice especially the reflux may be important as an etiological factor of the micropapillary carcinoma in this region (around pancreatic head).

On the other hand, invasive micropapillary carcinoma of the ampullo-pancreatobiliary region is often associated with tumor-infiltrating neutrophils [7]. In this case, numerous neutrophils were found around conventional adenocarcinoma (Fig. 2A) and there was mild eosinophilic infiltration around micropapillary component (not shown). The phenomenon (neutrophilic infiltration) was not reported in micropapillary carcinomas of other organs other than pancreas [7], and unexplained factors, such as chemotactic factors, may be responsible.

This case is bile duct cancer with small foci (2 mm in size) of micropapillary component. No reports have described the prognosis of cancer with such a small portion of micropapillary carcinoma. Because the presence of the minor micropapillary component may be ignored, carcinomas with a minor micropapillary component need to be carefully analyzed or followed up to understand their pathologic and prognostic characters.

## Conclusion

In summary, the first case of bile duct cancer with minor micropapillary component metastasizing to the regional lymph node as a micrometastasis is reported.

## Consent

Written informed consent was obtained from the patient for publication of this case report and accompanying images. A copy of the written consent is available for review by the Editor-in-Chief of this journal.

## Competing interests

The author declares that they have no competing interests.

## Authors' contributions

TK performed histological examination, analyzed the case, and wrote the manuscript.
